# Supplementary material for: Cross-species gut microbiota transplantation predictably affects host heat tolerance
Source: J Exp Biol. 2024 Jan 10;227(1):jeb246735. doi: 10.1242/jeb.246735 (PMC10906491; doi:10.1242/jeb.246735)
Supplement: Supplementary information [file jexbio-227-246735-s1.pdf]

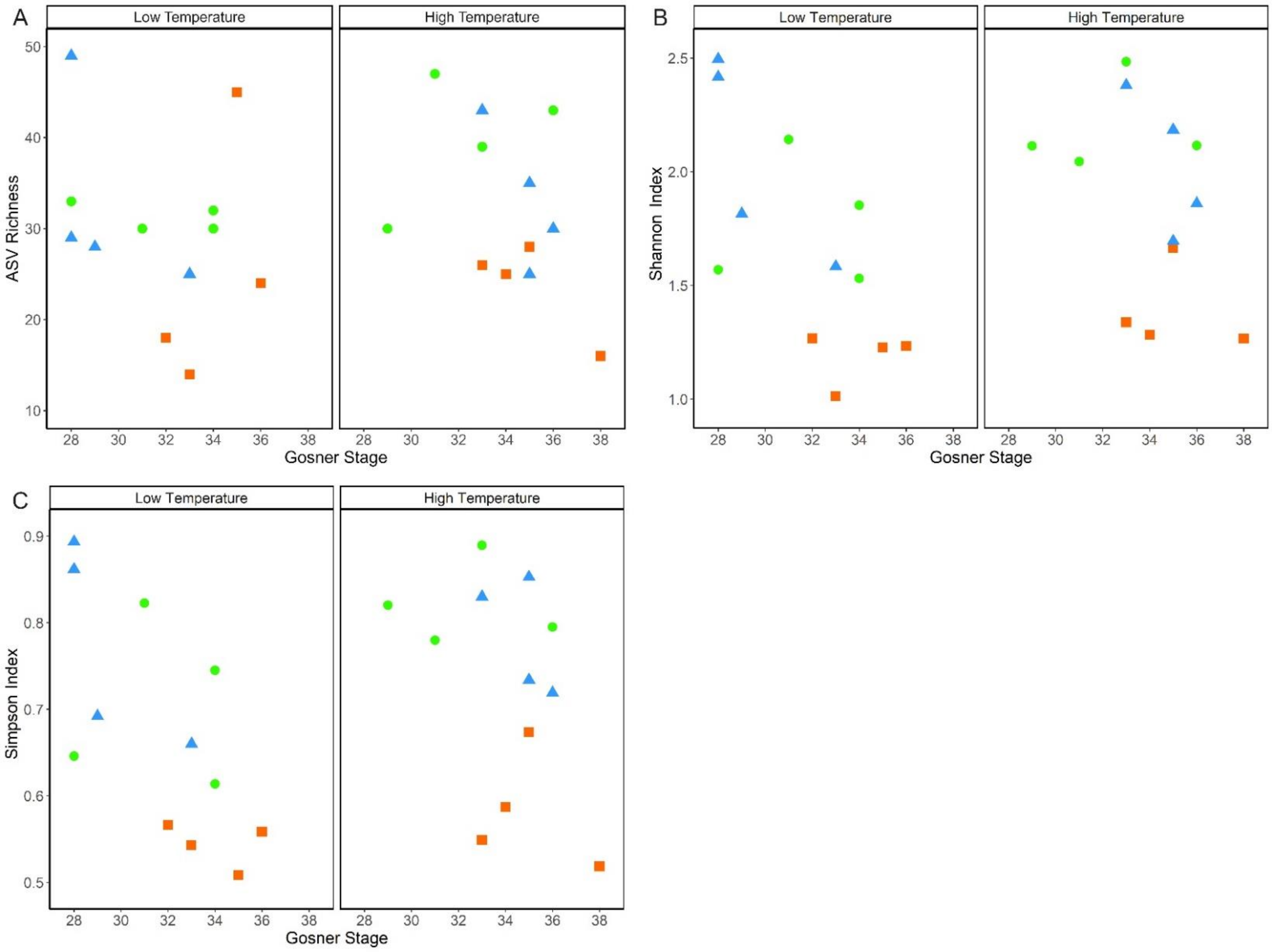

Fig. S1. The effect of gut microbiota treatments, acclimation temperature, and Gosner stage on alpha diversity indices of larval wood frogs: A) ASV Richness, B) Shannon Index, and C) Simpson Index. For both Shannon and Simpson indices, there was a statistically significant negative effect as alpha diversity was lower in more developed larvae. Sample sizes (n): Low Green Frog = 4, Low No Inoculum = 4, Low Wood Frog = 4, High Green Frog = 4, High No Inoculum = 4, High Wood Frog = 4.

Journal of Experimental Biology • Supplementary information

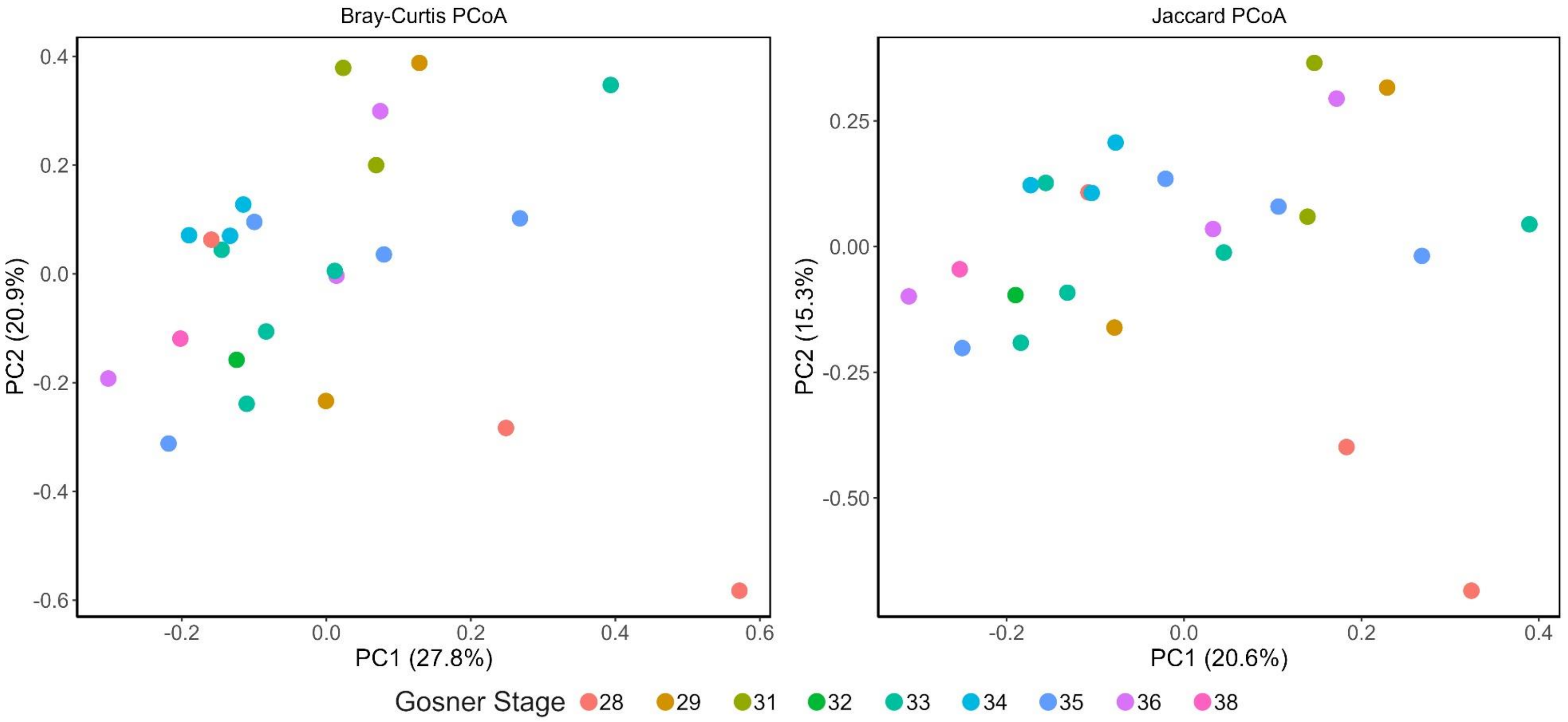

Fig. S2. Principal coordinate analyses (PCoA) of the gut microbiota community structure (A) and membership (B) of larval wood frogs separated solely by Gosner stage. Percentages on axes titles represent the variance explained by the eigenvector. Each point represents the gut microbiota of a wood frog larva. Sample sizes (n): Low Green Frog = 4, Low No Inoculum = 4, Low Wood Frog = 4, High Green Frog = 4, High No Inoculum = 4, High Wood Frog = 4.

Journal of Experimental Biology • Supplementary information

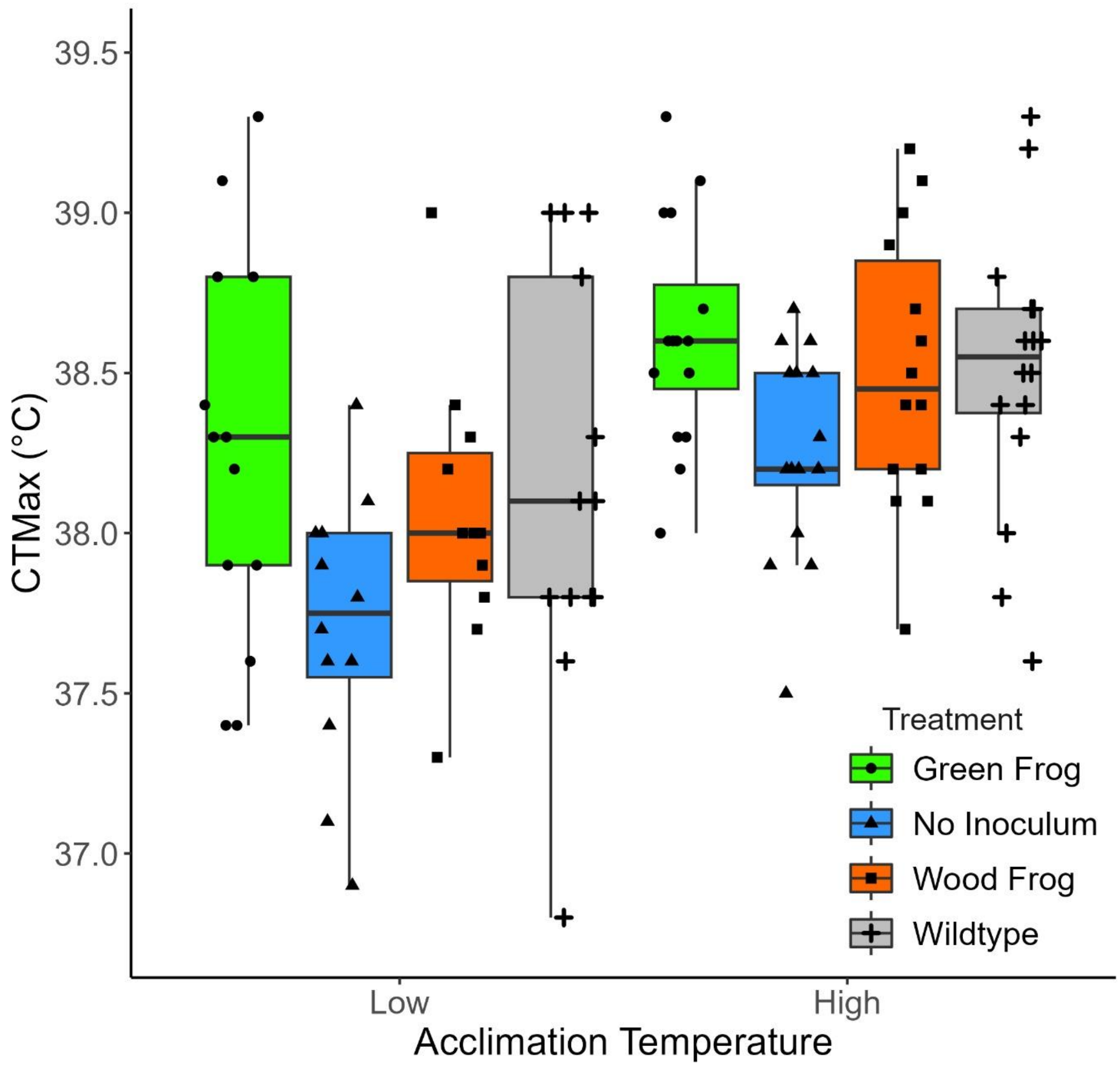

Fig. S3. The critical thermal maximum (CTmax) of larval wood frogs across all gut microbiota treatments and unmanipulated wildtype larvae. Center lines within boxplots represent the median and the boxes denote the interquartile range with whiskers representing 1.5× the upper or lower quartile. Sample sizes (n): Low Green Frog = 13, Low No Inoculum = 12, Low Wood Frog = 11, Low Wildtype = 13, High Green Frog = 16, High No Inoculum = 16, High Wood Frog = 14, High Wildtype = 16.

Journal of Experimental Biology • Supplementary information

Table S1. Relative abundances of bacterial phyla and families in tadpole gut microbial communities that were significantly impacted by acclimation temperature or gut microbiota treatment. Relative abundances are displayed as means ± 1 standard error. The group in which the specific taxa was most abundant is in bold. Statistical testing was conducted using MaAsLin2. N.O. represents groups where the bacterial taxa was not observed. P-values were corrected using the BH FDR method.

	**Relative Abundance (%)**			**BH FDR P-value**	**Coefficient**
**Temperature Effects**	**Low**	**High**			
**Family**					
Beijerinckiaceae	0.38 ± 0.15	**16.64 ± 2.42**		< 0.001	3.06
	**Relative Abundance (%)**			**BH FDR P-value**	**Coefficient**
**Microbiota Treatment Effects**	**Green Frog**	**No Inoculum**	**Wood Frog**		
**Family**					
[Clostridium] methylpentosum group	**0.52 ± 0.20**	N.O.	N.O.	< 0.001	–1.55
Clostridiaceae	< 0.01	**2.25 ± 0.87**	0.20 ± 0.08	< 0.03	
Rikenellaceae	**14.54 ± 2.65**	0.75 ± 0.50	0.06 ± 0.03	< 0.001	–4.21
Yersiniaceae	N.O.	**2.79 ± 1.26**	N.O.	< 0.001	2.93
Gracilibacteraceae	**1.00 ± 0.52**	N.O.	**0.67 ± 0.67**	< 0.01	–3.15
Aeromonadaceae	0.05 ± 0.05	**9.69 ± 3.06**	0.85 ± 0.83	< 0.01	3.45
Pseudomonadaceae	< 0.01	**2.20 ± 1.45**	0.07 ± 0.01	< 0.01	2.82
Flavobacteriaceae	0.01 ± 0.01	**1.61 ± 1.46**	**3.20 ± 1.42**	< 0.01	3.81
Promicromonosporaceae	**0.17 ± 0.07**	N.O.	**0.10 ± 0.07**	< 0.01	–1.52
Muribaculaceae	**0.08 ± 0.04**	N.O.	N.O.	0.01	–1.47
Uncultured Rickettsiales	**0.16 ± 0.12**	N.O.	N.O.	0.02	–1.24
Unknown Oscillospirales	**1.34 ± 0.57**	**3.78 ± 1.64**	0.06 ± 0.05	< 0.01	–4.24
Comamonadaceae	**1.01 ± 0.59**	**4.44 ± 1.86**	0.06 ± 0.04	< 0.01	3.58
